# Imaging performance of microscopy adaptive-optics system using scene-based wavefront sensing

**DOI:** 10.1117/1.JBO.25.12.123707

**Published:** 2020-12-16

**Authors:** Yusuke Ashida, Yusuke Honma, Noriaki Miura, Takatoshi Shibuya, Hayao Kikuchi, Yosuke Tamada, Yasuhiro Kamei, Atsushi Matsuda, Masayuki Hattori

**Affiliations:** a Kitami Institute of Technology, Kitami, Japan; b Utsunomiya University, School of Engineering, Utsunomiya, Japan; c Utsunomiya University, Center for Optical Research and Education, Utsunomiya, Japan; d National Institute for Basic Biology, Myodaiji, Okazaki, Japan; e Advanced ICT Research Institute, National Institute of Information and Communications Technology, Kobe, Japan; f National Astronomical Observatory of Japan, Tokyo, Japan

**Keywords:** adaptive optics, microscopy, scene-based wavefront sensing, image correlation

## Abstract

**Significance:** A scene-based adaptive-optics (AO) system is developed and a method for investigating its imaging performance is proposed. The system enables derivation of Strehl ratios from observed images via collaboration with computer simulations. The resultant Strehl ratios are comparable with those of other current AO systems.

**Aim:** For versatile and noninvasive AO microscopy, a scene-based wavefront-sensing technique working on a Shack–Hartmann wavefront sensor is developed in a modal control system. The purpose of the research is to clarify the imaging performance of the AO system via the derivation of Strehl ratios from observed images toward applications in microscopy of living cells and tissues.

**Approach:** Two imaging metrics that can be directly measured from observed images (i.e., an energy concentration ratio and unbiased maximum ratio) are defined and related to the Strehl ratio via computer simulations. Experiments are conducted using artificial targets to measure the imaging metrics, which are then converted to Strehl ratios.

**Results:** The resultant Strehl ratios are >0.7 and 0.5 in the cases of defocus and higher aberrations, respectively. The half-widths at half-maximum of the AO-corrected bead images are favorably comparable to those of on-focus images under simple defocus aberration, and the AO system works both under bright-field illumination and on fluorescent bead images.

**Conclusions:** The proposed scene-based AO system is expected to work with a Strehl ratio of more than 0.5 when applied to high-resolution live imaging of cells and tissues under bright-field and fluorescence microscopies.

## 1 Introduction

In biological microscopic observations, complex structures in the observation targets (e.g., nuclei, chloroplasts, and micro-oil drops) can result in wavefront errors. Thus, the observed images may be blurred.^1^^–^^3^ Adaptive optics (AO) can resolve such situations, and various AO systems have been developed for biological microscopy.^4^ A number of pioneering AO systems for microscopy have adopted indirect methods of phase-error measurement, including methods that use image metrics, and these have been successfully employed in biological applications.^4^ On the other hand, the direct wavefront-sensing technique has been used, specifically the one using a Shack–Hartmann (SH) sensor.^5^^,^^6^ With an SH sensor, the aperture of an optical system is divided into multiple subapertures (SA) using a microlens array, and replicas of the target image (i.e., SA images) are observed. The typical observation target of this sensor is a point-light source, and the overall wavefront phases can be obtained from all shifts of the point sources. The SH sensor consists of a simple optical system and enables robust and prompt measurement of phase errors, which is beneficial for biological microscopy. A serious problem, however, arises when the SH sensor is applied to biological imaging. In such a case, a proper point-light source is not always available.

To overcome this problem, Tao et al. used fluorescence from local excitation as a point-light source for SH-wavefront sensing, which was followed by similar research from Hattori and Tamada.^7^^–^^9^ However, attempts to introduce such artificial “guide stars” in living cells and tissues have been limited because of the necessity for strong fluorescence and undesirable photodamage to the target to a certain extent. Another promising method in which all fluorescence is accumulated during the point-scanning of a sample volume is proposed in which the integrated signal is then used for wavefront sensing.^10^^,^^11^ This method advantageously enables rapid and stable wavefront sensing with less damage. However, the optical set up of the system tends to be large scale.

In this study, another approach to solving the aforementioned problem is devised. Instead of a point-like guide star, nonpoint structures of the target are used for the SH-wavefront sensing. This technique has already been used as a correlation-based technique in solar AO^12^^–^^14^ and has gradually been recognized as a scene-based technique in biological microscopy.^15^^,^^16^ In such sensors, correlations between SA images are evaluated to detect their relative shifts. Notably, the scene-based technique can work under the condition that structures in the objects will appear on the SA images. Thus, additional optical components to the fundamental AO set up are not required.

In this research, a scene-based sensing technique is introduced into the microscopy AO system developed by Hattori and Tamada.^8^^,^^9^ Section 2 describes the details of this newly developed scene-based AO system. To investigate its imaging performance, microscopic observations are conducted on artificial testing targets developed by Hattori et al.,^17^ as discussed in Sec. 3. Fluorescence beads are used as objects in the artificial targets and imaging metrics that specify image quality from the observed bead images are derived. The purpose of this research is to clarify the relationship of the experimentally obtained imaging metrics with the Strehl ratio, which is frequently used in the evaluation of AO systems. The purpose is then to derive the Strehl ratios from the observed images. Although such ideas are important for estimating the true performance of AO systems,^5^^,^^6^ related studies have not been found despite the best efforts of the authors of this paper. In Sec. 4, computer simulations are designed and run to construct the relationships of the measured imaging metrics with the Strehl ratios. Finally, Sec. 5 summarizes this paper.

## 2 Microscopy AO System

**Fig. 1:**
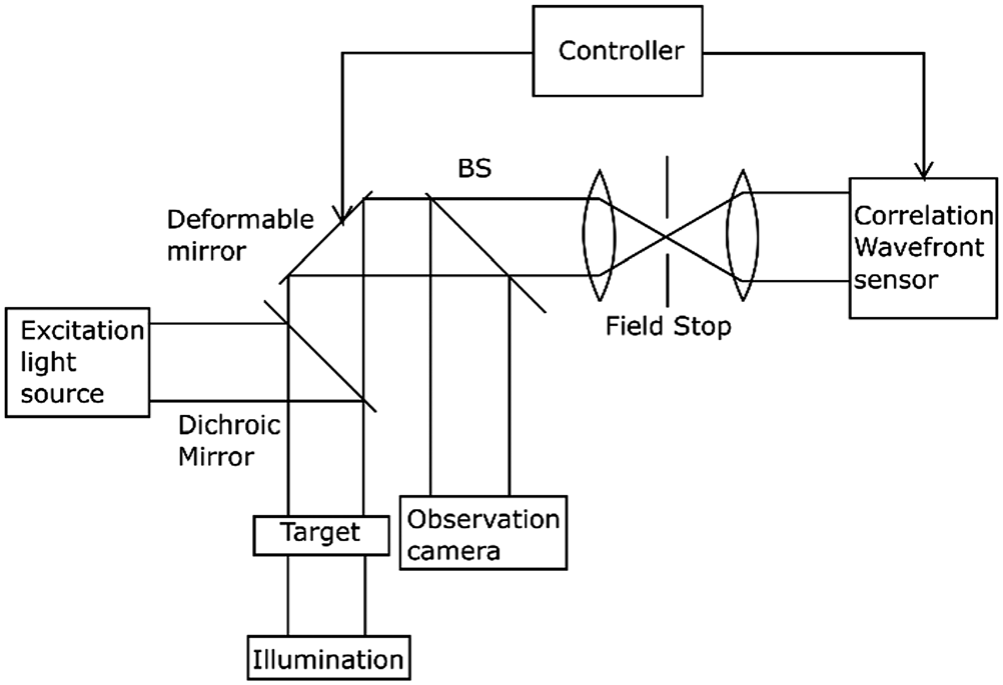
Configuration of the AO system. Wavefront sensor can be operated with nonpoint targets via a correlation-based algorithm for scene-based AO.

Figure 1 illustrates an AO system having a standard closed-loop configuration. A more detailed set up is available in the referenced literature.^8^ The system determines the wavefront phase errors using an SH-wavefront sensor. Then it controls a deformable mirror (DM), which is based on micro-electro-mechanical systems (Boston Micromachines, Multi-DM), to correct the phase errors. To achieve effective correction, these two steps must be repeated in real time. The controller is an AO software developed by the authors and runs on a standard personal computer. The type of illumination can be switched between excitation light and bright-field illumination according to observational requirements. Calibration of the optical set up and parameter tuning are performed using a pinhole on the sample plane under bright-field illumination.

### 2.1 Wavefront Sensor

**Fig. 2:**
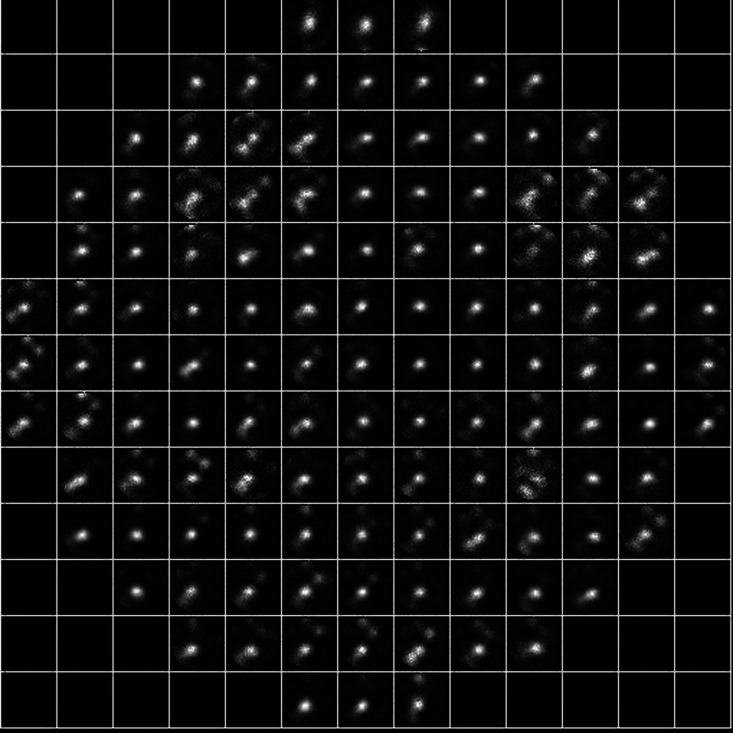
Image of fluorescent beads (nonpoint objects) obtained using scene-based SH-wavefront sensor. Each subimage is shifted because of local wavefront tilts at SA position. Relative shifts of these subimages are detected via a correlation algorithm.

The SH-wavefront sensor used in the experiments consists of a 13×13 microlens array (Thorlabs, MLA150-5C, f=4.1  mm, 150-μm pitch) and a scientific complementary metal-oxide-semiconductor camera (Hamamatsu Photonics, C11440-22CU). As shown in Fig. 2, images captured by the camera have multiple SA subimages. The central 121 of the 169 SA subimages covering the effective area of the DM are used for the calculation. If the wavefront incident to the sensor is plain, each SA image will be positioned at its origin. When the wavefront is perturbed, the positions of the SA images will deviate from the origin in proportion to the local gradients of the wavefront. Therefore, the wavefront phase errors can be calculated from the shifts of the SA images. A major difference between the proposed technique and standard SH-wavefront sensing is the adoption of image correlations in the measurement of image shifts, as described in the following section.

### 2.2 Wavefront Estimation Using Image Correlation

**Fig. 3:**
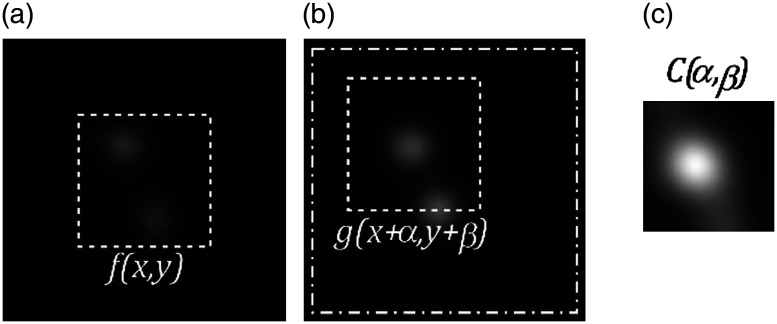
Correlation-based measurement of image shifts for nonpoint target with two beads. (a) Central part of one SA image (dashed line) is used as reference pattern f(x,y) for correlation. (b) Another SA image g(x,y) is correlated with f(x,y) scanned within the dash-dot square to obtain distribution of correlation values C(α,β) as shown in (c). White denotes maximum. Shift amount is evaluated as position of maximum value.

Let one SA be indicated as a reference for correlation in advance. The central part of the reference SA is then used as a reference pattern, f(x,y), as shown in Fig. 3(a). In the following experiments, the reference SA is set to the center SA after visually confirming that it does not include local aberrations.

On another SA image g(x,y), we calculate the image correlation

$$C(\alpha ,\beta )=\frac{\sum \sum f(x,y)g(x+\alpha ,y+\beta )}{\sqrt{\sum \sum f^{2}(x,y)}\sqrt{\sum \sum g^{2}(x+\alpha ,y+\beta )}},$$ (1)

which is repeated for different values of (α,β) [Fig. 3(b)]. The position (α,β), where the correlation value is maximum, is identified [Fig. 3(c)] and used on the shift amounts resulting from the wavefront phase errors. A shift vector s with shift amounts as elements is determined for all SAs.

The wavefront phases ϕ(u,v) can be expanded as

$$\phi (u,v)=\sum_{j=1}^{J}a_{j}Z_{j}(u,v),$$ (2)

where $Z_{j}(u,v)$ is the jth Zernike polynomial and $a_{j}$ is its coefficient. Because the shift amounts are proportional to the local gradients of ϕ(u,v), the shift vector can be related to them in matrix form as

s=Za, (3)

where Z is a matrix with the partial derivative values of the Zernike polynomials, and a is a vector of the coefficients. The inverse matrix of Z is calculated in advance and a is determined via multiplication by the shift vector s.

**Fig. 4:**
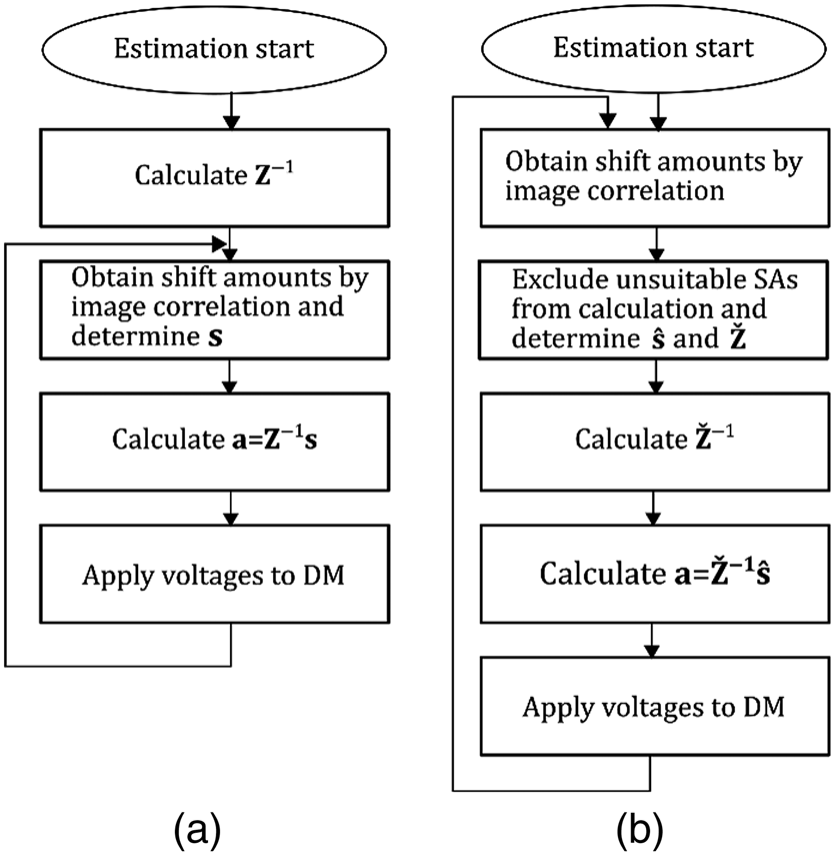
(a) Procedure of Zernike modal wavefront correction used in conventional AO system. Calculation of $Z^{-1}$ is performed once. By contrast, $\overset{ˇ}{Z}$ calculations (b) are performed in every loop of the proposed microscope AO.

To obtain the Zernike coefficients correctly, it is important to accurately measure the shift amounts. Because living biological tissues frequently result in locally strong aberrations, a number of the observed SA images are severely deformed. As a result, the accuracy of the estimation is deteriorated. This problem is resolved via exclusion of the SAs in which the detection of the correct shift amounts is difficult. This exclusion reduces the number of columns of s and Z, producing their subsets, s^ and $\overset{ˇ}{Z}$, respectively. If the number of polynomials is very high, reduction by a few columns will hardly affect the precision of estimation. Figure 4 compares the procedures of wavefront correction with and without SA exclusion. Matrix Z in Eq. (3) is calculated once before the AO correction loop in the conventional algorithm, whereas matrix $\overset{ˇ}{Z}$ is recalculated every time in the loop when the system performs an exclusion of the SA images. In the software used for this study, the SAs to be excluded can be designated on the graphical interface by the user prior to starting the AO and can be fixed during the AO loop.^16^

In the following experiments, SA exclusion is not applied because the aim is to estimate the best performance of the system, which is expected to occur when all SAs are used. Comparisons of image qualities may be interesting in cases where all SAs are used under strong local aberrations and where some locally degraded SAs are excluded, despite the tradeoff from the reduction in the number of SAs. However, these issues are beyond the scope of this study and will be explored in future experiments using living cells and tissues.

## 3 Imaging Performance Under Various Conditions

### 3.1 Defocus Aberration

**Fig. 5:**
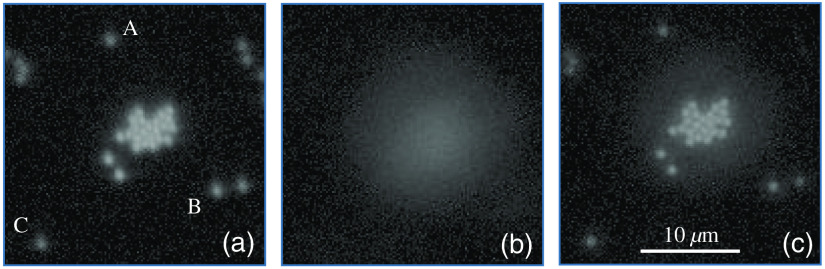
Images of fluorescence beads (a) on focus, (b) intendedly defocused, and (c) corrected with AO. Images are displayed with γ=2 to emphasize dark parts.

The first experiment was conducted to test AO performance under a low-order aberration. The target was fluorescence from microbeads (Thermo Fisher Scientific, G0100) excited by cyan light-emitting-diode light, as shown in Fig. 5(a). The beads were 1.0-μm in diameter. Between the beads and the microscope objective were silicone oil (n=1.427), a coverslip, and air. The microscope objective was an Olympus Plan N 40× (NA=0.65). The imaging camera was a Thorlabs DCU223M with 1024×768  pixels and 8-bit gray level. The noise levels in the observed images were measured by calculating the root-mean-squared errors over the region of no object. The value was σ=2.62, which is used later to derive errors in the imaging metrics.

Defocus phase errors were intentionally introduced by the movement of the microscopic stage in the z direction. As expected, the observed image was severely blurred, as shown in Fig. 5(b). Starting from the defocused state, the AO system was operated to obtain the image in Fig. 5(c). It was restored to a fine state in (a), but slightly darker. Additionally, halo components appeared around the beads. Generally, point-spread functions (PSF) through AOs comprise a sharp peak (core) and its surrounding components (halo).^5^ Beads located near the edges in (c) are noticeably darker than those around the center. Light emitted from them is partly obscured by the edges of the optical components, because the stage was moved in the z direction to induce defocus.

**Fig. 6:**
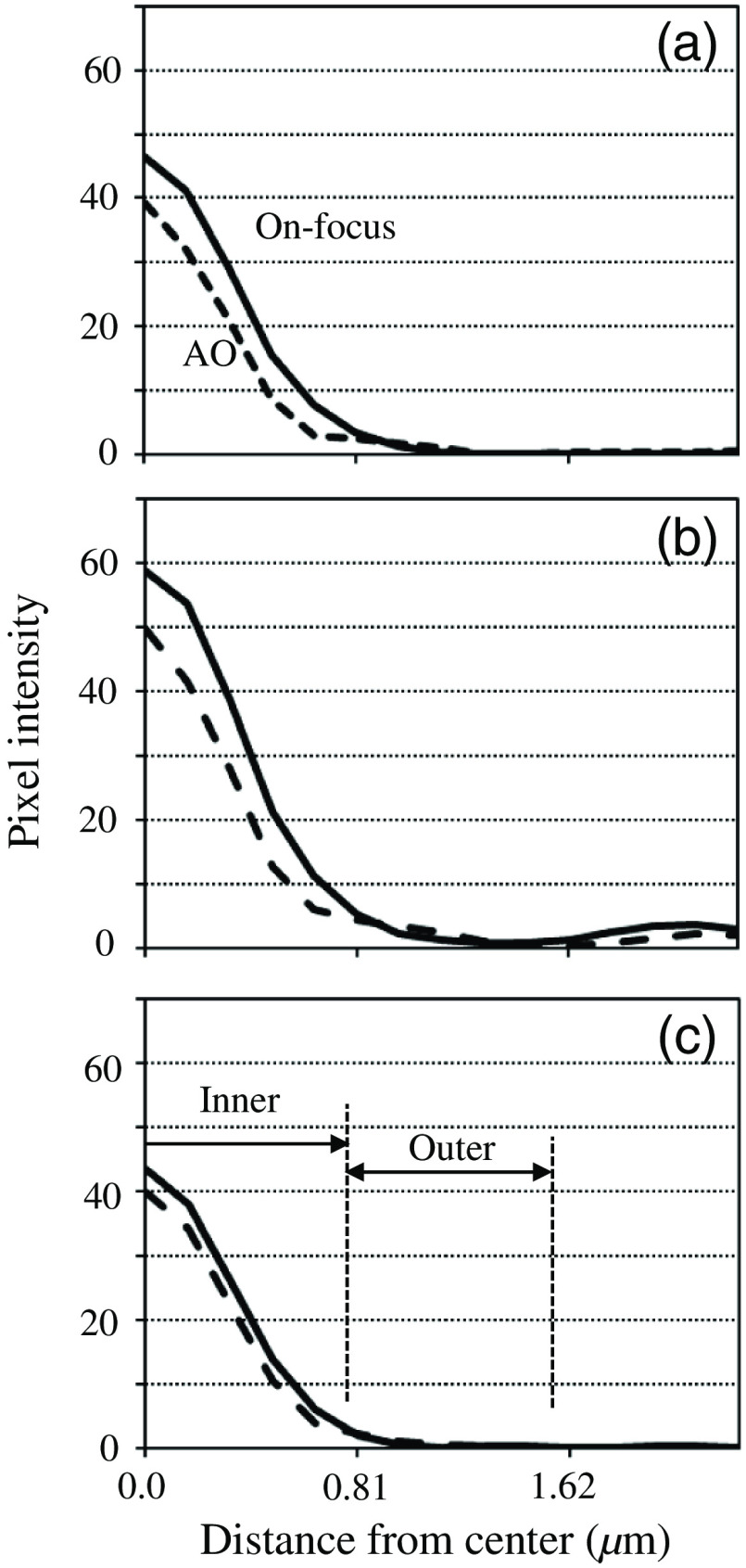
Intensity profiles of (a)–(c) beads A–C on images: focused (solid) and AO-corrected (dashed).

Three isolated beads, A, B, and C, in Fig. 5(a), were chosen for the analysis of imaging performance. Figures 6(a)–6(c) show the profiles of radial averages around the centers of beads A–C. The subpixel position and the peak value were derived by parabolic approximation using pixel values around the maximum. The solid and dashed lines correspond to the images observed in focus and after AO correction, respectively. Every plot shows the same tendencies. That is, the intensities of the central peaks became smaller after AO, whereas those of the halos were barely larger. The half-widths at half-maximum (HWHM) were calculated from the profile using linear interpolation. As shown in Table 1, the HWHMs in the AO-corrected image were slightly narrower than those in the on-focus image. The AO is inferred to have been able to compensate for not only the large defocus component but also the small aberrations induced by the structure of the artificial testing target. The silicone oil (n=1.427) of 30-μm thickness filled between the fluorescent beads and the coverslip (n=1.52) of 0.13 to 0.16 mm in the artificial target and fairly matched the specifications of the microscope objective (0.17-mm-thick coverslip in air). However, there was a small gap of the refractive indices between layers of the silicon oil and the cover slip, causing beam refraction.

**Table 1:** HWHM of bead images (μm).

Beads	On-focus image Fig. 5(a)	AO-corrected image Fig. 5(c)
A	0.39±0.02	0.34±0.01
B	0.41±0.01	0.36±0.01
C	0.38±0.02	0.36±0.02

In this experiment, the Zernike polynomials were used from the 4th to the 21st term. Their coefficients, listed in Table 2, were derived using the SH sensor. The huge defocus component of 10.15 rad, intentionally induced with the stage shift, was reduced to −0.10 rad through AO correction. Additionally, the value of the spherical aberration before AO 0.22 was relatively high, which may have been caused by the mismatch of the objective and the coverslip as described previously. It was substantially reduced to −0.05 after AO. Through the reconstruction of the wavefront phase pattern from the Zernike coefficients measured before AO correction, the Fried parameter $r_{0}$ was directly calculated via the determination of the maximal radius of a circle, inside which the deviation of phase errors was <1  rad. The result was $D/r_{0}=3.13$, where D is the diameter of the aperture. The value $D/r_{0}$ denotes the number of turbulent cells aligning on the aperture diameter, frequently used to indicate the degree of image degradation. The residual wavefront error after AO correction was $\sigma_{\mathrm{AO}}=0.16\;\mathrm{rad}$, theoretically yielding a Strehl ratio, $r_{\mathrm{ST}}=\exp(-\sigma_{\mathrm{AO}}^{2})$ of 0.98.^5^ This is the same level reported by Tao et al.^7^

**Table 2:** Zernike coefficients measured using developed wavefront sensor (rad).

Aberration	Before correction	After correction
Defocus	10.15	−0.10
Vertical astigmatism	−0.01	0.00
Oblique astigmatism	0.15	0.01
Horizontal coma	−0.09	−0.01
Vertical coma	0.03	−0.01
Vertical trefoil	−0.06	−0.01
Oblique trefoil	0.08	0.01
Spherical	0.22	−0.05
Second vertical ast.	0.01	0.01
Second oblique ast.	0.03	0.00
Vertical quadrafoil	0.03	0.06
Oblique quadrafoil	0.03	0.01
Second horizontal coma	0.10	−0.05
Second vertical coma	−0.06	−0.03
Second vertical trefoil	0.05	−0.04
Second oblique trefoil	−0.01	0.03
Vertical pentafoil	−0.06	−0.04
Oblique pentafoil	−0.10	−0.01

However, Strehl ratios measured from the observed images ought to be lower than those derived from residual phase errors on the sensor. One reason is the noncommon optical path from the DM to either the sensor or the observation camera. Therefore, phase errors arising from optical defects would not be perfectly removed, even if all Zernike coefficients measured on the sensor became zero. The other reason is the limited number of SAs, Zernike polynomials, and DM actuators. Higher-order wavefront aberrations still remain, even after AO correction. Therefore, it is necessary to derive Strehl ratios from the observed images to fairly evaluate the AO system.

For this purpose, to estimate the proportion of energy from a given bead concentrate inside a certain patch, an energy concentration (EC) ratio was introduced. An inner circle having a radius of 0.75  μm, which is the sum of the bead radii and the HWFM of the Airy pattern, and an outer ring having inner and outer radii of 0.75 and 1.50  μm, respectively, were set on each bead image [Fig. 6(c)]. With the total pixel values inside the inner circle and the outer ring, $T_{\mathrm{circ}}$ and $T_{\text{ring}}$, respectively, the EC ratio was defined to be $r_{\mathrm{EC}}=T_{\mathrm{circ}}/(T_{\mathrm{circ}}+T_{\text{ring}})$. Most of the energy accumulates inside the inner circle of an ideal optical system, whereas the deterioration of a PSF decreases the EC ratio because of decreases in the core intensity and increases in the halo level. The EC ratio is useful as a metric for evaluating imaging performance because it can be converted to the Strehl ratio, as shown in Sec. 4.

Table 3 summarizes the results. The EC ratios in the on-focus image were 0.86 to 0.92. The variety in these values may have arisen from inhomogeneity in the sizes and shapes of the light patterns emitted from the beads. The errors in the EC ratio were specific to the noise level of the images. Precise EC values could not be derived from the defocused image because it was very dark. However, when AO was used, EC values of 0.77 to 0.87 were attained. As can be seen in Fig. 6(b), the profiles of bead B might contain some excessive components leaked from its surrounding bead. These excessive components contributed to the lower EC ratio. As a result, the Strehl ratios after AO were 0.73 to 0.92. The values inside the brackets in Table 3 show the ranges of the Strehl ratios. For example, when the EC ratio was 0.89±0.04, the lower and upper limits of the Strehl ratios were converted to 0.85 and 0.93, respectively. However, 0.93 exceeded the maximum value (0.92), resulting in a Strehl ratio of 1.0. In this case, the EC ratio was truncated to the maximum value.

**Table 3:** EC ratios of bead images and corresponding Strehl ratios.

Beads	On-focus image Fig. 5(a)				AO-corrected image Fig. 5(c)			
	$T_{\mathrm{circ}}$	$T_{\text{ring}}$	$r_{\mathrm{EC}}$	Strehl ratios	$T_{\mathrm{circ}}$	$T_{\text{ring}}$	$r_{\mathrm{EC}}$	Strehl ratios
A	1,158	142	0.89±0.04	0.96 [0.88, 1.00]	733	188	0.80±0.04	0.79 [0.71, 0.86]
B	793	128	0.86±0.04	0.90 [0.83, 0.98]	690	210	0.77±0.03	0.73 [0.68, 0.79]
C	1,019	88	0.92±0.05	1.00 [0.92,1.00]	821	128	0.87±0.05	0.92 [0.83, 1.00]

This section is thus concluded: the HWHMs of the AO-corrected bead images were comparable to those of the on-focus images. However, the EC ratios after AO correction decreased to 89% to 94% of those for the on-focus images. This deterioration might have been caused by wavefront errors remaining after the AO, including higher-order components, which could not be corrected by the developed system. Strehl ratios of 0.73 to 0.92 are regarded as the maximum limit in AO performance of the system.

### 3.2 Higher-Order Aberrations

**Fig. 7:**
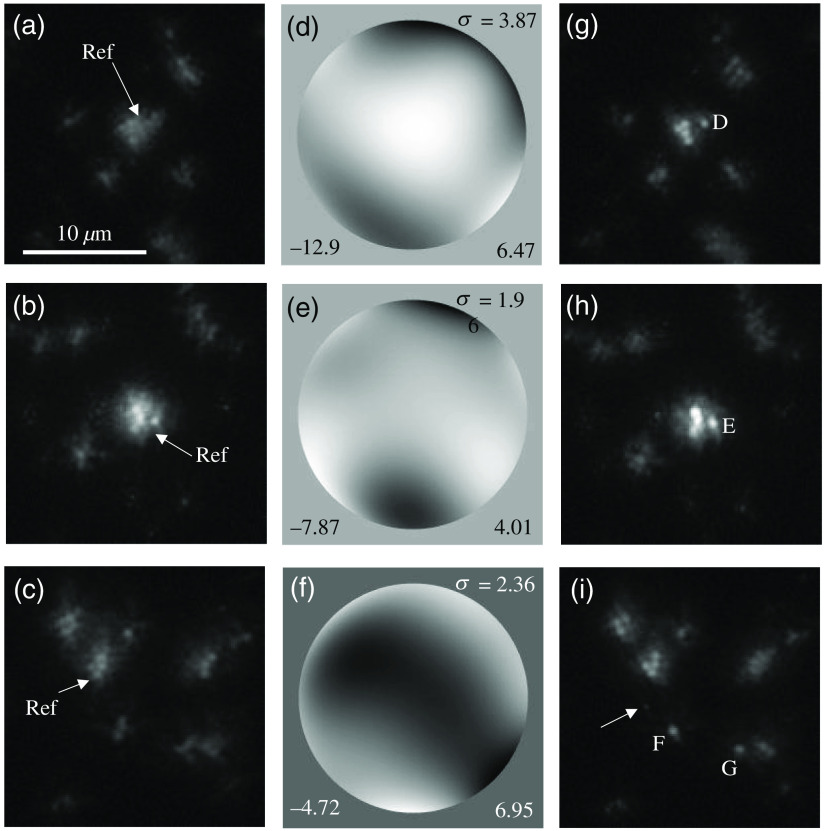
(a)–(c) Blurred images of fluorescence beads on different positions of the artificial testing target. (d)–(f) Wavefront phases measured by wavefront sensor when images (a)–(c) are acquired. White and black denote maximum and minimum, respectively. (g)–(i) Results of AO correction of (a)–(c), respectively. Values on (d)–(f) show wavefront phases in radians: deviation (upper), minimum (lower left), and maximum (lower right). Beads D–G are used for analyses. Label “Ref” indicates the reference pattern used for wavefront sensing.

The following experiments were performed using an artificial testing target made by Hattori et al.,^17^ which can be used as a model for a moss leaf (*Physcomitrella patens*).^18^ Objects in the target included a mixture of fluorescent beads with diameters of 1.0 and 0.37  μm. An etched coverslip was used to induce higher-order phase errors of approximately half the wavelength of 530 nm. Through changes in the positions on the target via shifting of the microscopic stage, the AO correction experiments were repeated. Figures 7(a)–7(c) show images observed at different positions of the artificial testing target. The spherical shapes of the beads are hardly recognizable, especially in (a).

**Fig. 8:**
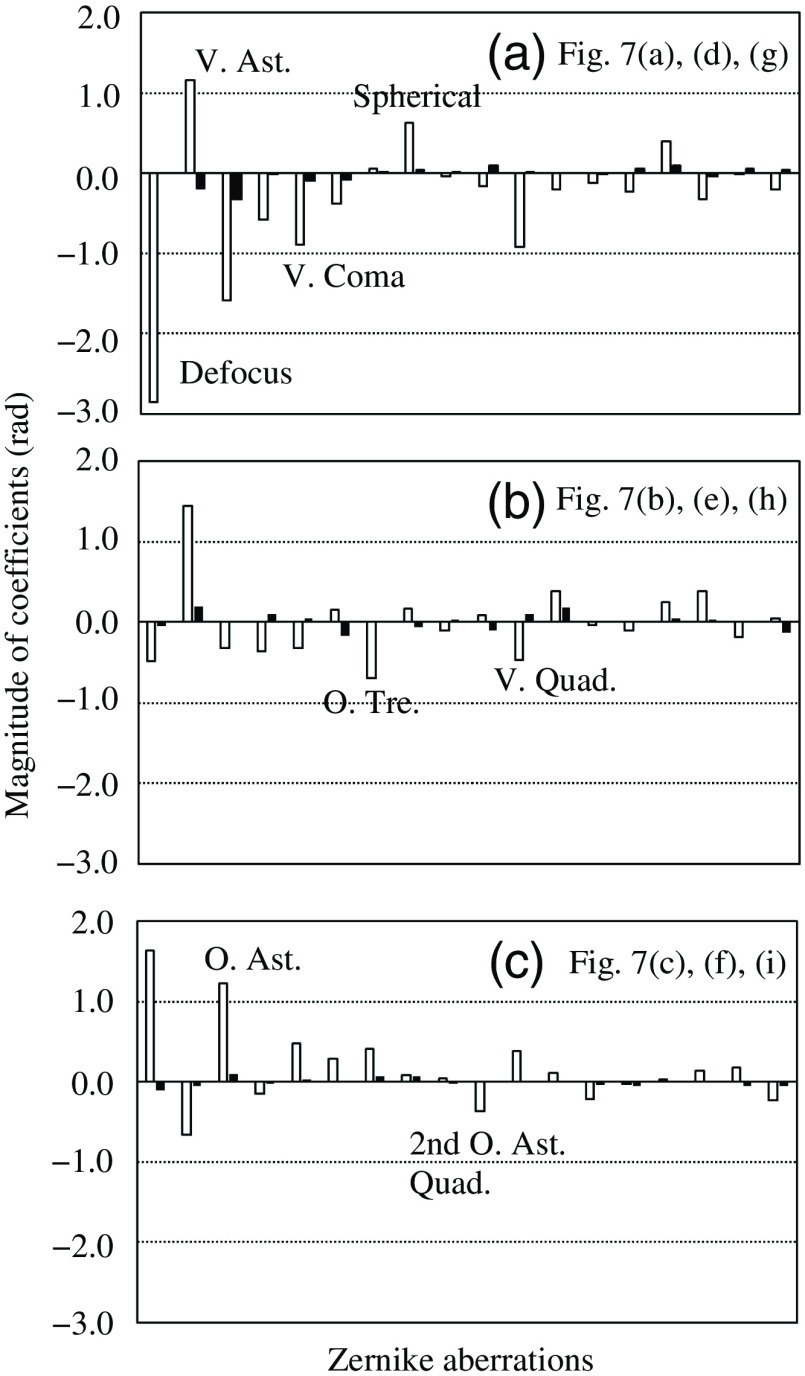
Zernike coefficients measured with wavefront sensor. (a)–(c) correspond to images on top, middle, bottom rows in Fig. 7. Open and filled bars show magnitudes of coefficients before and after AO corrections, respectively. Order of Zernike aberrations is same as that in Table 3.

The Zernike coefficients were simultaneously recorded while the bead images were being acquired and are shown as open bars in Fig. 8. It should be reemphasized that true wavefront phases can involve higher-order components than what was measured by the sensor. Figures 7(d)–7(f) show the wavefront phases reconstructed from the Zernike coefficients as gray images. The phase patterns can be observed to change position by position on the artificial testing target. The Fried parameter $r_{0}$ was calculated in the same manner as previously described. The results are as follows: (d) $D/r_{0}=2.62$, (e) 1.64, and (f) 1.84. Although these wavefront errors were not very large, the images of (a)–(c) appear to contain a noticeable levels of foggy components. Parts of the foggy components may have arisen from the diffusion of light occurring on the etched surface of the coverslip, where about half the wavelength of depth enhanced the diffractive errors.^2^^,^^17^

Figures 7(g)–7(i) show the AO-corrected images. As expected, bead images of 1.0-μm diameter around the reference patterns became finer such that their individual shapes were distinguishable as being spherical. Moreover, a bead image of 0.37-μm diameter appeared, as indicated by the arrow in (i), although it was not distinguished prior to AO correction. At image regions apart from the reference pattern, the effect of restoration declined and finally became zero. This is because the wavefront errors induced by the etched coverslip varied because of its position, an effect known as anisoplanatism.^5^ The Zernike coefficients after AO are also shown as filled bars in Fig. 8. These were greatly reduced compared with those before AO, shown as open bars in Fig. 8. The deviations in wavefront errors before correction were (a) 3.87, (b) 1.96, and (c) 2.36 rad, as displayed in Figs. 7(d)–7(f), respectively, whereas these values became 0.44, 0.40, and 0.22 rad after correction.

**Fig. 9:**
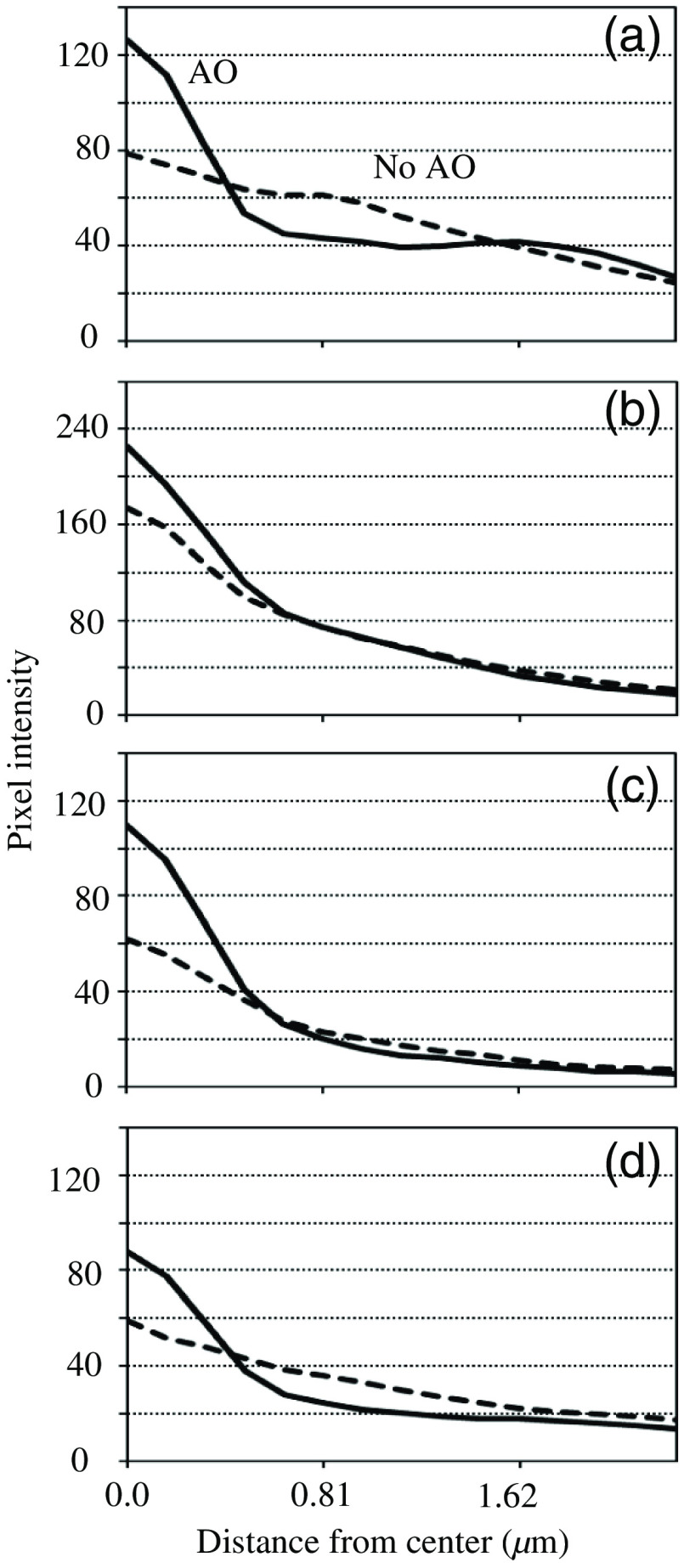
Intensity profiles of (a)–(d) beads D–G on images: blurred (dashed) and AO-corrected (solid).

Relatively isolated beads were chosen from Figs. 7(g)–7(i) and labeled as D–G. Their profiles are shown in Fig. 9. The fluorescence intensity was more concentrated in the cores by the application of AO. However, the curves of beads D and E were derived via azimuthal averaging with respect to not the entire range, but to only the right half, to prevent the curves from being affected by the left bright beads. Because bead F was nearly isolated, its profile was regarded as not exhibiting any effects from the other beads. Nevertheless, it obviously had more bias components compared with Fig. 6(c). These bias components may have been caused by diffusion, as mentioned previously. The other profiles had more excessive bias components because there were other beads close to the target. Therefore, HWHM was calculated only for bead F. The HWHMs derived were 0.63±0.02 and 0.40±0.01  μm before and after correction, respectively. The value was noticeably reduced by the AO, but was slightly higher than those in Table 1. This may have been caused by the effects of bias components.

To derive the Strehl ratio for profile having bias components, an unbiased maximum (UM) ratio was introduced. Generally, as shown in Fig. 9, the profile curves with and without AO cross because the AO heightens the core and lowers the halo. With the pixel value at the cross point used as base value, the UM value was defined as (maximum value) − (base value). The radius of the cross point was determined via computer simulation, as described in Sec. 4. The pixel value at the radius was derived from the profile curve and was subtracted from the maximum value to obtain the UM value. Through subtraction, the effects of the bias components can be reduced or perfectly removed if the bias is flat. UM values $M_{\mathrm{ao}}$ and $M_{\mathrm{no}}$ were calculated from the profiles measured with and without AO, respectively. Then their ratio, $r_{\mathrm{UM}}=M_{\mathrm{no}}/M_{\mathrm{ao}}$, was obtained. The relationship between the UM ratio and the Strehl ratio was clarified using computer simulation, and the UM ratio was determined to be available for some situations (see Sec. 4).

Table 4 summarizes the results. The errors in the UM ratios are specified together with both the noise levels of the images and the errors caused by parabolic approximations in the derivation of the maximum values. All Strehl ratios were 0.52 to 0.77. This range of values indicates that the AO system could attain a Strehl ratio of more than 0.5 when applied to the microscopic observation of moss leaves. Azucena et al.^19^ estimated the Strehl ratios expected for their sample after correcting 14 Zernike components, determining the mean value of the ratio to be 0.597. The results in this study, which uses scene-based wavefront sensing, are comparable.

**Table 4:** UM ratios and corresponding Strehl ratios.

Beads	$M_{\mathrm{no}}$	$M_{\mathrm{ao}}$	$r_{\mathrm{UM}}$	Strehl ratios
D	15.29	75.31	0.20±0.03	0.52 [0.48, 0.58]
E	73.43	111.77	0.67±0.03	0.59 [0.58, 0.60]
F	25.85	69.76	0.37±0.03	0.70 [0.67, 0.74]
G	16.10	49.94	0.32±0.04	0.77 [0.71, 0.84]

### 3.3 Bright-Field Illumination

**Fig. 10:**
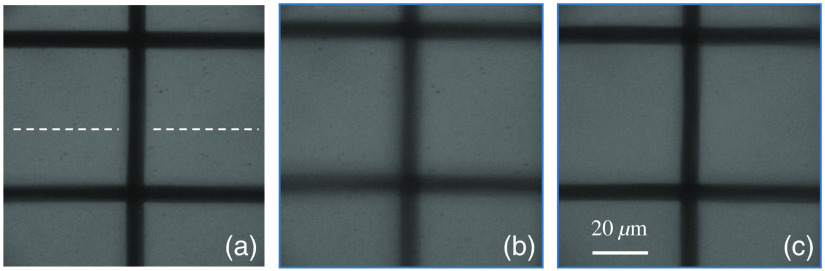
Images of grid under bright-field illumination (a) on focus, (b) intendedly defocused, and (c) corrected with AO.

**Fig. 11:**
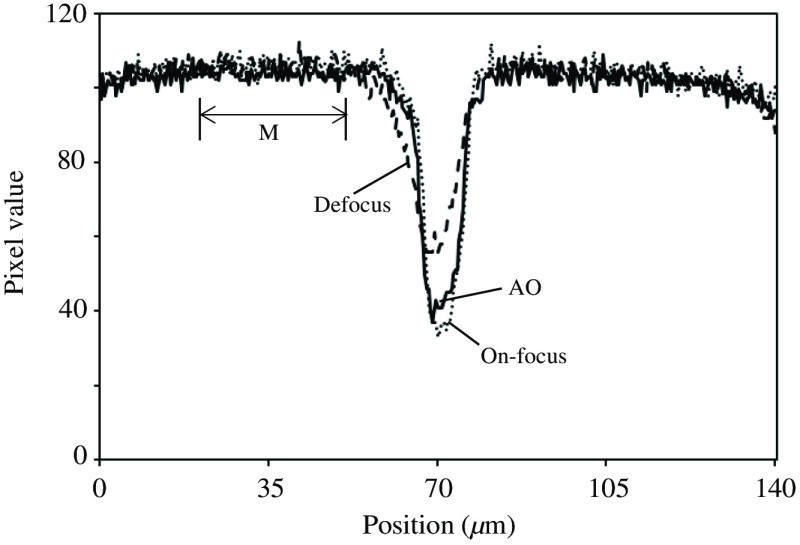
Intensity profiles of grid images along dashed lines in Fig. 10.

The following experiment was performed under bright-field illumination using a grid having intervals of 50  μm (Thorlabs, R1L3S3P). Zernike polynomials were used from the 4th to the 21st terms. Figures 10(a) and 10(b) show the grid images observed at the focus and defocus positions, respectively. The AO system was started from the defocused state to obtain the grid image shown in Fig. 10(c). The reference for wavefront sensing is represented by the lower cross pattern. Figure 11 shows the profiles of the grid images along the horizontal lines, as indicated by the dashed lines in Fig. 10(a). Through AO correction, the improvement in image quality was obvious. The full-widths at half-maximum (FWHMs) of the brightness dip by the vertical grid lines were measured. The FWHMs were 8.16, 10.64, and 8.72  μm, respectively. For the maximum value in the FWHM calculations, the mean value over the region labeled “M” in Fig. 11 was adopted. The contrast of the grid was also calculated to be (max−min)/(max+min). The results were 0.52 (on-focus), 0.30 (defocus), and 0.47 (AO). Both the FWHM and contrast improved through the AO, but they were slightly worse than those of the on-focus image. This was caused by residual phase errors after the AO decreased the PSF cores and increased the intensity of PSF halos.

Table 5 lists the Zernike coefficients measured using the scene-based wavefront sensor. As expected, the values were greatly reduced by the AO correction. The deviation of wavefront errors was 8.46 rad before AO correction, whereas it dropped to 0.17 rad afterward. This accuracy is comparable to the result of the first experiment despite having different levels of illumination.

**Table 5:** Zernike coefficients measured under bright-field illumination (rad).

Aberration	Before correction	After correction
Defocus	8.17	−0.06
Vertical astigmatism	−1.42	0.13
Oblique astigmatism	0.03	−0.03
Horizontal coma	0.25	0.04
Vertical coma	−0.85	0.04
Vertical trefoil	−0.79	−0.01
Oblique trefoil	0.41	−0.04
Spherical	0.00	−0.03
Second vertical ast.	0.18	0.00
Second oblique ast.	0.44	−0.01
Vertical quadrafoil	−0.47	0.00
Oblique quadrafoil	0.00	0.00
Second horizontal coma	0.39	−0.03
Second vertical coma	−0.17	−0.04
Second vertical trefoil	0.05	−0.01
Second oblique trefoil	0.03	0.04
Vertical pentafoil	0.64	0.00
Oblique pentafoil	−0.43	−0.01

## 4 Computer Simulations

### 4.1 Emitting Pattern of Beads

The purpose of the computer simulations was to enable interpretation of the imaging metrics obtained from the Strehl-ratio experiments when using models of observations on the fluorescent microbeads. PSFs through AOs generally comprise cores having diffraction-limited components and halos that are widely spread around the cores. The shape of the core is a well-known Airy disk pattern denoted as A(x). For the halo, the Gaussian function H(x) has an HWHM of

$$x_{\mathrm{HW}}=0.61f_{\mathrm{ob}}(\lambda /D)/{\left[1+{\left(D/r_{0}\right)}^{2}\right]}^{1/2},$$ (4)

where λ is the wavelength and $f_{\mathrm{ob}}$ is the focal length of the objective used.^5^ Thus the PSF in the AO observations is defined as

P(x)=εA(x)+(1−ε)H(x), (5)

where ε is a parameter specifying the degree of AO correction: ε=1 or 0, signifying either full or no correction, respectively. The functions A(x) and H(x) are normalized such that the total intensity becomes unity. The Strehl ratio is defined as $r_{S}=P(0)/A(0)$. If the AO correction is perfect, then ε=1 and $r_{S}=1$ will be attained.

**Fig. 12:**
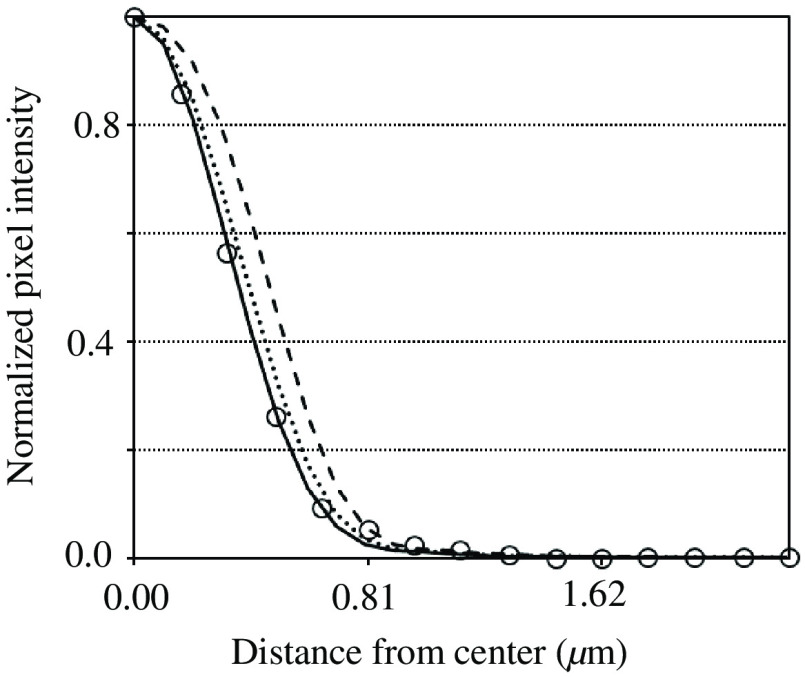
Intensity profiles of bead images when the emitting pattern of the bead is assumed to be (a) uniform (dashed curve), (b) spherical (dotted curve), and (c) parabolic (solid curve). Measured values (open circles) fit best with the solid curve.

The emitting pattern of fluorescence B(x) from the beads used as objects in the experiments should be identified next. Three emitting patterns were assumed: (a) uniform disk B(x)=1; (b) half-sphere $B(x)={\left[1-{\left(|x|/r_{\max}\right)}^{2}\right]}^{1/2}$; and (c) parabola $B(x)=1-{\left(|x|/r_{\max}\right)}^{2}$; where $|x|≦r_{\text{max}}$, and $r_{\text{max}}$ is the radius of the beads (0.5  μm). B(x) was convolved with P(x) to produce a simulated observed image and its intensity profile along the x axis was obtained. Figure 12 shows the three intensity profiles when ε=1. The normalized values of the measurements of an on-focus bead C are also plotted, similar to what is shown in Fig. 6(c). The curve of the (c) parabola clearly fits most of the measurements. Therefore, a parabolic shape was adopted as the model of the emitting pattern of the bead.

### 4.2 Energy Concentration Ratio versus Strehl Ratio

**Fig. 13:**
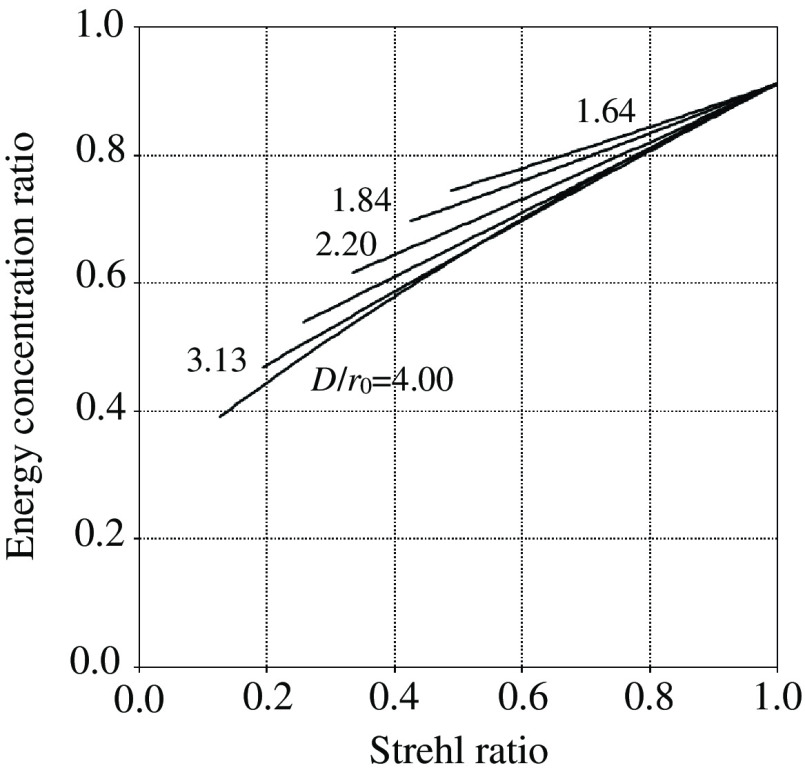
Relationship of EC ratio with Strehl ratio for different values of $D/r_{0}$.

After the emitting pattern B(x) of the bead was identified, convolving it with P(x) yielded a simulated bead image. From the bead image, the total intensities inside the inner circle and outer ring, $T_{\mathrm{circ}}$ and $T_{\text{ring}}$, respectively, could be easily calculated, leading to $r_{\mathrm{EC}}=T_{\mathrm{circ}}/(T_{\mathrm{circ}}+T_{\text{ring}})$. The value of ε was changed from zero to unity $r_{S}$ and $r_{\mathrm{EC}}$ were executed, and the values were plotted. These executions were repeated using different values of $D/r_{0}$. In the experiments, $D/r_{0}=1.64$, 1.84, 2.62, and 3.13 were the values actually encountered, whereas $D/r_{0}=2.20$ and 4.00 were used to fill gaps between parameters. As shown in Fig. 13, the relationships were almost linear for lower $D/r_{0}$, whereas the plots became slightly curved as $D/r_{0}$ increased. Additionally, the curves tended to be shorter in cases of smaller $D/r_{0}$. This indicates that the Strehl ratio does not become small even when the effect of AO is low, because PSFs having small $D/r_{0}$ have narrower halo components with higher center values. Such differences in the features are treatable under conditions where the value of $D/r_{0}$ is known. Therefore, the EC ratio is concluded to be useful for evaluating image quality, leading to the Strehl ratio.

The conversion of the EC ratio to the Strehl ratio can be numerically performed if $D/r_{0}$ is known. By changing ε in the software code, both EC and Strehl ratios are calculated under the specified $D/r_{0}$. When the former is best fitted to the measurement of the EC ratio, the latter is adopted as the estimation of the Strehl ratio.

### 4.3 Unbiased Maximum Ratio versus Strehl Ratio

**Fig. 14:**
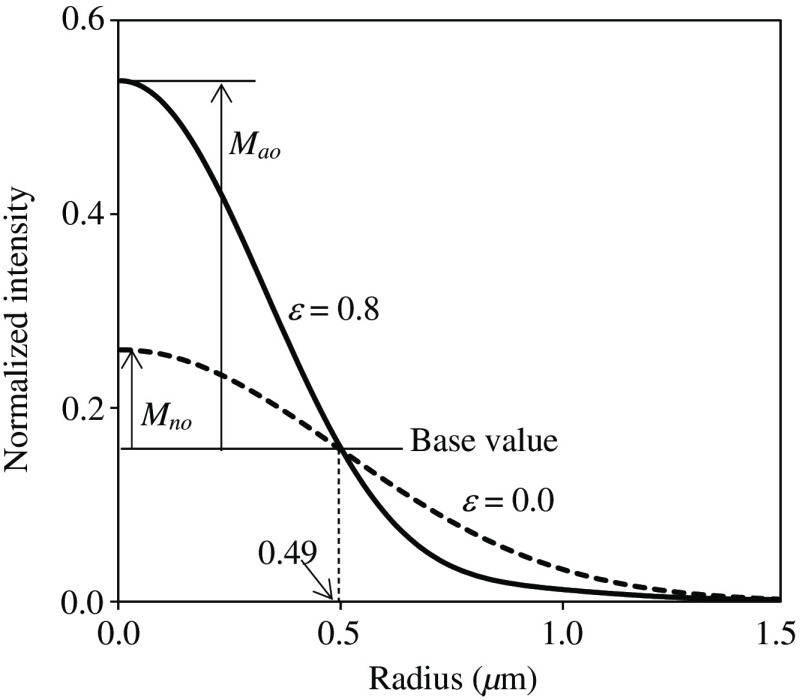
Illustration of UM values when $D/r_{0}=1.84$.

In the experiment described in Section 3.2, the UM ratio was introduced to estimate system performance despite the existence of bias components on bead images. Figure 14 shows the profiles of the bead images when defocused (ε=0) and AO-corrected (ε=0.8) when $D/r_{0}=1.84$. Obviously, the two profiles cross at a point and they have the same value there. This is referred to as the base value. The UM values $M_{\mathrm{ao}}$ and $M_{\mathrm{no}}$ are indicated in the figure. Advantageously, as described, the UM value is unchanged, even when the profiles contain flat bias components. The radius of the cross point was checked, thus changing its parameters. The radius was confirmed to vary dependently with $D/r_{0}$, but remained unchanged with respect to ε. The radii obtained were 0.48, 0.49, 0.52, 0.55, 0.59, and 0.63  μm for $D/r_{0}=1.64$, 1.84, 2.20, 2.62, 3.13, and 4.00, respectively.

**Fig. 15:**
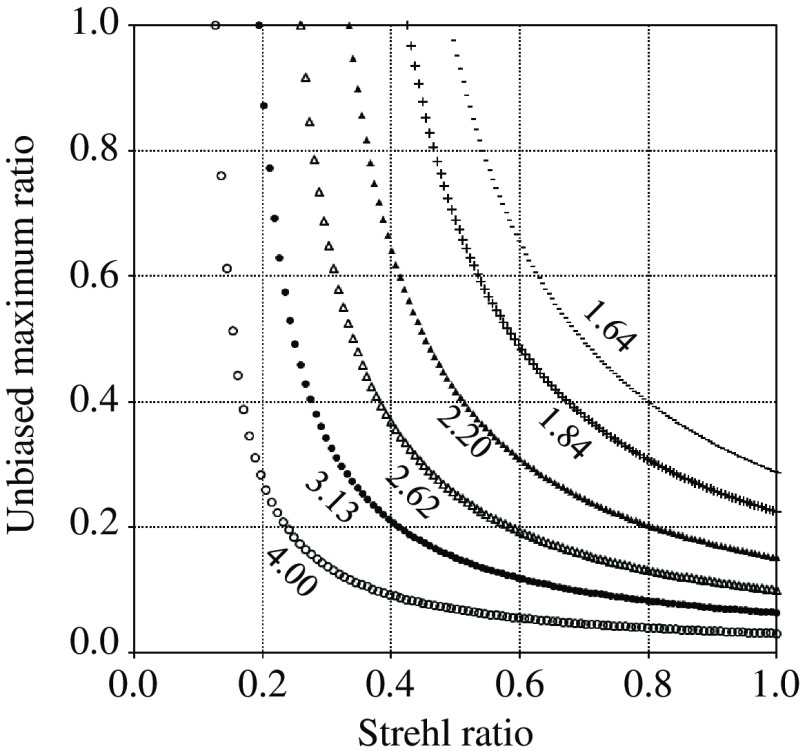
Relationship of UM ratio with Strehl ratio.

Identical to the previous simulation, the relationships of $r_{\mathrm{UM}}$ with $r_{\mathrm{ST}}$ for various $D/r_{0}$ were obtained. The results are shown in Fig. 15. The curves were more bent because the values of $D/r_{0}$ were larger and almost flat at the larger parts of the Strehl ratios. In these ranges, the Strehl ratios entail large errors when converted from the UM ratios. Therefore, the UM ratios used for conversion should be larger than the threshold in which the absolute value of the gradient is 0.5. Consequently, the error amplification was suppressed to less than double during the conversion of the UM ratio to a Strehl ratio. The threshold values were 0.32, 0.29, 0.24, 0.20, 0.16, and 0.12 when $D/r_{0}=1.64$, 1.84, 2.20, 2.62, 3.13, and 4.00, respectively. Because the UM values derived in the experiments were not less than the thresholds, the Strehl ratios listed in Table 4 were derived.

The conversion of the UM ratio to the Strehl ratio can be done as follows. The radius of the cross point is determined from $D/r_{0}$. The base value at the cross point and the maximum value at the center is measured from a bead profile. Then their difference is taken to be a UM value. Repeating those using the bead profiles before and after AO and taking the ratio of UM values derived yields the measurement of an UM ratio. It should be noted that the effects of bias components can be removed though the difference operation. Similar to the previous section, when changing ε in the software code, both UM and Strehl ratios are calculated under the specified $D/r_{0}$. When the former is best fitted to the measurement, the latter is adopted as the estimation of the Strehl ratio.

## 5 Summary

The proposed microscope AO system was described in detail using image correlations in SH-wavefront sensing. Experimental results were presented to confirm the performance of the AO system under bright-field and fluorescence microscopies.

The first experiment measured the performance of the AO system when only a large defocus component existed. From the measurements of the HWHMs of bead images, a resolution equivalent to an on-focus observation was confirmed by the AO. Additionally, the EC ratio was defined and leveraged to evaluate each bead image. The resulting EC ratios following AO correction were 89% to 94% of the on-focus image. These values were converted to Strehl ratios ranging from 0.73 to 0.92. These values are regarded as the expected maximum performance of the proposed system.

The second experiment was performed to test AO performance for images blurred having higher-order aberrations. An artificial testing target that modeled a moss leaf was used to evaluate AO performance when applied to the observation of living specimens. From the experimental results, the bead images were observed to have been improved but slightly expanded compared with those of the first experiment. That is, the resolutions worsened by ∼10%. This may have been caused by bias components in the observed images related to diffusion. The UM ratio was also defined and it was found to be useful in limited cases of both smaller $D/r_{0}$ and larger UM ratios. When the UM ratios obtained in the experiments were converted, Strehl ratios ranging from 0.52 to 0.77 were obtained. These values were at the same level as reported for another AO system.

The last experiment was the same as the first except for object and illumination. Notwithstanding the differences, the proposed AO system was confirmed to have attained the same level of wavefront compensation. This result suggests that the new AO system can be used independently of illumination type.

From these results, the proposed scene-based AO system is expected to work with a Strehl ratio >0.5 when applied to the high-resolution live imaging of cells and tissues under bright-field and fluorescence microscopies. The next step is to confirm its use on living specimens.
